# Local Anæsthesia

**Published:** 1854-12

**Authors:** 


*Local Anaesthesia.* (*Translated from the Journal de Medecine et de Chirurgie Pratiques, for the Charleston Medical Journal and Review.*)

Experiments upon local anæsthesia are still continued both in France and England, and we do not consider ourselves too sanguine, in expressing a well grounded hope that sensibiliiy may, after a while, be locally diminished and suspended, by impregnating in some way, the external and accessible organs with vapour of chloroform. We have unfortunately not witnessed ourselves any thing decisive in this matter as yet: but in England it appears that results have been more favorable. Two important communications have been addressed to the Medico-Chirurgical Society of London, and to the Surgical Society of Ireland, which show that in these places experiments upon this subject are being rapidly pushed forward, and that a large number of physicians have already obtained very remarkable results. Thus Mr. Snow has discovered that lint (charpie) impregnated with chloroform, placed upon the skin, and covered over with a bit of oiled silk, occasions at first a degree of burning in the part; after a few moments, sensibility becomes diminished and the skin may be transfixed with pins without pain; but on pursuing his investigations, he has found that the epidermis opposes itself more or less to the anaesthetic influence, and that when it was previously removed, insensibility of the dermis was very easily determined, and it could be pierced and lacerated with impunity.

This observation is of great importance to the success of local anaesthesia; it is, moreover, in conformity with the experiments of other surgeons, and of Mr. Hardy in particular, upon *currents of anaesthetic vapors directed upon the mucous surfaces.* The effects upon these vapor *douches* have been very remarkable, and we cannot avoid making known a few of the observations communicated to the Surgical Society of Ireland by this latter physician.

We have already spoken of the instrument of Mr. Hardy, imported into France by Charriere, and by means of which the surgeon of Dublin injected anaesthetic vapors into the vagina, rectum and bladder. It is with this instrument that he has continued his experiments, and he is now in possession of a number of facts, conclusively showing the efficacy of this measure in circumstances of very varied character. Other surgeons have followed his example; thus, Dr. O'Reilly has communicated to the Society of Obstetrics many remarkable facts, and among others, the following:—A poor woman was suffering from a cancer of the rectum opening into the vagina and causing intolerable pain, against which large doses of opium had been administered. The vapor of chloroform directed upon the part, produced an instantaneous relief. It was then practicable to clean and dress the cancerous surface, a thing which its extreme sensibility had before rendered quite impossible, and this practice being continued, the patient soon found herself so much more comfortable that she was able to rise and reappear in society. An insufflation of the vapor into the rectum during fifteen or twenty minutes, sufficed to obtain abundant repose, and it was specially used for this purpose repeatedly. During three months these fumigations with chloroform have been continued, and have in no wise lost their efficacy. The patient finds herself quite improved from its use, and since its first employment there has been no necessity to have recourse to opium or any other narcotic.

Another observation, due to Dr. Ringland, is no less remarkable. A child, but two months old. was affected with a bronchite, attended with suffocation and extreme agitation, and had not slept a moment for seventy-two hours. A great number of remedies had been tried, but without the least amelioration. At length, a small quantity of the vapor of chloroform was injected into the rectum by the process of Mr. Hardy. After an interval of less than a quarter of an hour, the child became calm, and fell into a profound sleep, which lasted for two hours. When it awoke, it took the breast, which it had constantly refused before its sleep. The employment of the anaesthetic vapors was resumed and shortly afterwards the child fell once more into a deep sleep. It was necessary to awaken it after several hours to give it the breast. This process was repeated several times and conducted to a complete cure.

A few days since, says Mr. Hardy, a physician was speaking to me of a woman who was suffering from a violent pain in the ear. She was unable to procure a moment of repose, notwithstanding the use of numerous remedies. Finally it was resolved to apply a *douche* of chloroform to the meatus. After the lapse of ten minutes the pain ceased, and the patient slept profoundly. The following day she was troubled with a return of the pain, which, however, yielded immediately to a renewed application of the vapor. Mr. Hardy has observed a case precisely similar; the patient had introduced into the meatus, some cotton steeped in chloroform, without other effect than a sensation of heat. The same substance employed in vaporous *douches*, calmed the pain entirely after about eight or ten minutes, and no further uneasiness was experiened.

In a case of tetanus, a couple of these instruments have been used, to apply the douche to both masseters at once, and in a short time it was possible to open his mouth.

But M. Hardy has not restricted himself to the application of these douches to small surfaces; he has invented an apparatus which emits these vapors, either with or without admixtnre of warm water, in such a manner to maintain in contact with them a large surface of the skin at a time. An aneurism of the popliteal artery first suggested to him the propriety of modifying the instrument which had so well succeeded upon a small scale and upon surfaces of a limited extent. The tumour in this case was treated by compression, and the pain resulting from the process was such that the patient was utterly unable to a low the bandaging to remain in situ. M. Hardy then bethought himself of an instrument constructed upon larger proportions than the first. By its means veritable vapor baths can be administered, nd if it be desirable to act with vigor, a whole member, the pelvis, abdomen, or chest can be entirely enveloped. M. Hardy promises himself important results from the application of these vapors of chloroform, in a host of affectioi s now happily modified by means of poultices, fomentations, embrocations, &c., methods far less powerful than the anaesthetic vapous, associated or not with water or other medicates.

Many members of the society have also declared that these vapor, douches had very well succeeded with themselves. and one makes mention of the ablation of a finger, without pain to the patient.

If we may judge of the matter, by the two communications above cited, this meʇɥod is teceived with much favor in England, and we may soon expect more important results.

